# Design and Performance of Extraordinary Low-Cost Compact Terahertz Imaging System Based on Electronic Components and Paraffin Wax Optics

**DOI:** 10.3390/s22218485

**Published:** 2022-11-04

**Authors:** Vincas Tamošiūnas, Linas Minkevičius, Ignotas Bučius, Domas Jokubauskis, Karolis Redeckas, Gintaras Valušis

**Affiliations:** 1 Institute of Photonics and Nanotechnology, Vilnius University, Saulėtekio Ave. 3, LT-10257 Vilnius, Lithuania; 2 Department of Optoelectronics, Center for Physical Sciences and Technology (FTMC), Saulėtekio Ave. 3, LT-10257 Vilnius, Lithuania

**Keywords:** terahertz imaging, terahertz detectors, high-electron-mobility transistors

## Abstract

Terahertz (THz) imaging is a powerful technique allowing us to explore non-conducting materials or their arrangements such as envelopes, packaging substances, and clothing materials in a nondestructive way. The direct implementation of THz imaging systems relies, on the one hand, on their convenience of use and compactness, minimized optical alignment, and low power consumption; on the other hand, an important issue remains the system cost and its figure of merit with respect to the image quality and recording parameters. In this paper, we report on the design and performance of an extraordinary low-cost THz imaging system relying on a InP Gunn diode emitter, paraffin wax optics, and commercially available GaAs high-electron-mobility transistors (HEMTs) with a gate length of 200 nm as the sensing elements in a room temperature environment. The design and imaging performance of the system at 94 GHz is presented, and the spatial resolution in the range of the illumination wavelength (∼3 mm) and contrast of nearly two orders of magnitude is determined. The operation of two models of the HEMTs of the same nominal 20 GHz cut-off frequency, but placed in different packages and printed circuit board layouts was evaluated at 94 GHz and 0.307 THz. The presence of two competing contributions—self-resistive mixing and radiation coupling through the antenna effects of the printed circuit boards—to the detected signal is revealed by the signal dependence on the gate-to-source voltage, resulting in a cross-sectional responsivity of 27 V/W and noise-equivalent power of 510 pW/$\sqrt{\mathrm{Hz}}$ at 94 GHz. Further routes in the development of low-cost THz imaging systems in the range of EUR 100 are considered.

## 1. Introduction

The ability of terahertz (THz) radiation to penetrate through non-conducting or dielectric materials provides it with an essential role in the development of nondestructive imaging systems and a large-scale of different applications ranging from material probes, security systems, and industrial inspection to bio- or medical diagnostics [1,2]. The direct implementation of THz imaging systems, from the scientific point of view, is strongly dependent on their convenience of use and compactness, minimized optical alignment, and low power consumption and, from a commercialization point of view, on the costs of the THz instrumentation, which is rather expensive currently and thus requires a cost reduction. As a rule, THz imaging systems are made up of three main constituents: radiation sources, passive optical elements, and the detection segment. Pioneering imaging experiments employed either optically pumped molecular THz lasers [3] or femtosecond-laser-based optoelectronic THz emitters [4], i.e., relatively bulky radiation sources. Electronic multipliers are more compact and less power consuming and, thus, can be more convenient in THz imaging experiments, particularly in the sub-THz range [5,6]. The next step in THz emitter miniaturization can be attributed to the handling of quantum cascade lasers [7] in imaging systems; however, due to the requirements of effective operation at room temperature, only intracavity mixing schemes in quantum cascade structures [8,9] can be a rational way to proceed in the direct implementation as the highest operating temperature using conventional cascade generation schemes can reach a maximum of 250 K currently [10]. A compact and low-priced solution in passive optics can be realized by replacing the bulky parabolic or spherical mirrors with relevant diffractive optic components [11] or metamaterial-based optical elements [12]. There are several compact sensing approaches widely used for room temperature imaging. We emphasize bolometric sensing devices [13,14,15,16]; Schottky diodes [17,18] and bow-tie sensors [19,20], enabling direct, spectroscopic, and real-time THz imaging applications. However, in the recent decade, high-electron-mobility transistors (HEMTs) have received much attention due to their high sensitivity, frequency tunability, flexibility in design, and wide integration ability [21,22,23,24]. More specifically, detectors based on silicon field effect transistors (FETs) and HEMTs based on III–V technology are actively developed as devices that can combine high sensitivity and fast response times [25]. Recently, a voltage responsivity of $R_{v}=15$ kV/W and a minimal noise-equivalent power of NEP = 0.58 pW/Hz^0.5^ were demonstrated at room temperature at 0.14 THz using 250 nm gate-length AlGaN/GaN HEMTs coupled with nanoantennas on a Si substrate [26].

It is worth noting that, in addition to specialized FET-based transistor sensor designs, commercial off-the-shelf transistors have also attracted considerable attention. Despite the fact that they are usually developed for radio frequency (RF) signal amplification, these FETs can successfully be applied for THz sensing. For example, polarization-sensitive detection of 100 GHz radiation was demonstrated employing the Fujitsu FHX45X HEMT, which was designed for signal amplification within 2–18 GHz frequencies [27]. It was revealed that the origin of the detected signal can be attributed to the interplay of excited 100 GHz currents, the influence of the bonding wires, and substrate-related modes. The importance of resonances related to bonding wires and metallic pads was exposed by detailed investigations of the photoresponse spectra obtained using the Avago ATF-36077 pseudomorphic HEMT (pHEMT) [28].

In this work, the design and performance of an extraordinary low-cost THz imaging system consisting of a InP Gunn diode emitter-based electronic source delivering radiation power of 42 mW at 94 GHz, a paraffin wax lens, and commercially available 200 nm gate-length GaAs HEMTs are demonstrated. The detected signal arises from two competing contributions—self-resistive mixing and radiation coupling through the antenna effects of the printed circuit boards, exhibiting a cross-sectional responsivity of 27 V/W and an NEP of 510 pW/$\sqrt{\mathrm{Hz}}$ at 94 GHz. The system displays a spatial resolution in the range of the radiation wavelength (∼3 mm) and an image contrast of nearly two orders of magnitude. Two models of the HEMTs detectors of the same nominal 20 GHz cut-off frequency, but in different packages and printed circuit board layouts were evaluated for comparison at 94 GHz and 0.307 THz. For the direct implementation, in addition to the recording parameters, an important issue remains the system cost and its figure of merit with respect to the image quality. Since the emitter accounts for less than EUR 3000, the paraffin wax lens does not exceed EUR 1, and the price of the 20 GHz pHEMT transistors serving as the sensor are of the order of EUR 1/unit for larger batches, it opens an optimistic route for an essential reduction in the price of THz imaging systems with reasonable image recording parameters.

## 2. Materials and Methods

**Figure 1:**
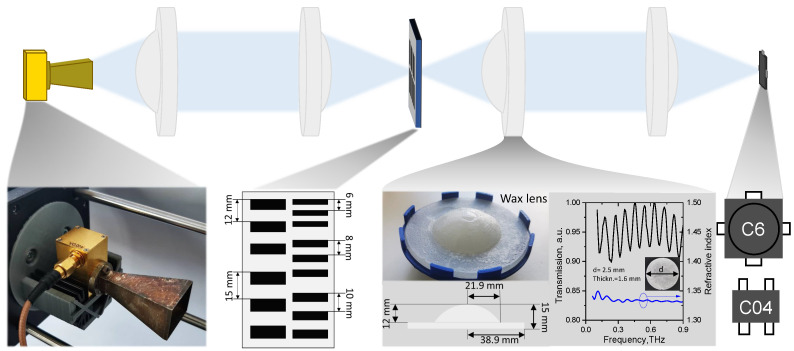
Schematic of the experimental setup and photos of the setup’s components with their parameters. Upper panel: As the THz radiation source serves the InP Gunn diode delivering radiation of 94 GHz in frequency with a power of 42 mW, 3D-printed paraffin wax lenses are used to collimate and focus the THz radiation, and CE3521M4 transistors operate as the THz detectors. Bottom panel: The photo of the Gunn diode, the parameters of the target produced from aluminum foil; photo of the paraffin wax lens, its geometrical parameters, and the dispersion curve of the refractive index; the composition of the layouts of the printed circuit boards of the investigated HEMTs.

The THz imaging experiment was performed on the set-up shown in Figure 1. The radiation of 42 mW at 94 GHz delivered by the InP-based Gunn diode in free space was collimated by the paraffin wax lens with a focal length of 50 mm. The refractive index of the purchased paraffin wax used for lens fabrication and its dispersion was determined using a frequency domain spectrometer based on a low-temperature-grown gallium arsenide (LTG GaAs) photomixer coupled with a silicon lens (Teravil, UAB, Vilnius, Lithuania). The lens of 26 mm in radius was fabricated using a material-extrusion-based 3D printer—Ultimaker 2 with an xy position precision of 12.5 µm and 5 µm in the *z* direction. Since 94 GHz corresponds to a wavelength of 3 mm, the obtained roughness in the lens shape of 0.1 mm has no essential influence on its focusing performance. Lens parameters and its refractive index dispersion are depicted in the bottom panel of Figure 1.

The beam from the 94 GHz source was collimated and then focused on the target using two lenses. The target shown in Figure 1 was custom-made from thin aluminum foil, which had grating periods varying from 5 mm to 15 mm. The radiation passing through the target was collimated once again and then focused on the HEMT detector.

Two models of 20 GHz pHEMT transistors produced by California Eastern Laboratories (CEL) were used in the experiments, CE3520K3 and CE3521M4. One needs to note that these pHEMTs possess the same typical saturated drain current, gate-to-source cut-off voltage, and transconductance, suggesting the same or at least a very similar processing sequence of the semiconducting parts of the devices. At the same time, the packages and external contact layouts are different openings, thus the possibility of investigating different coupling with THz radiation.

**Figure 2:**
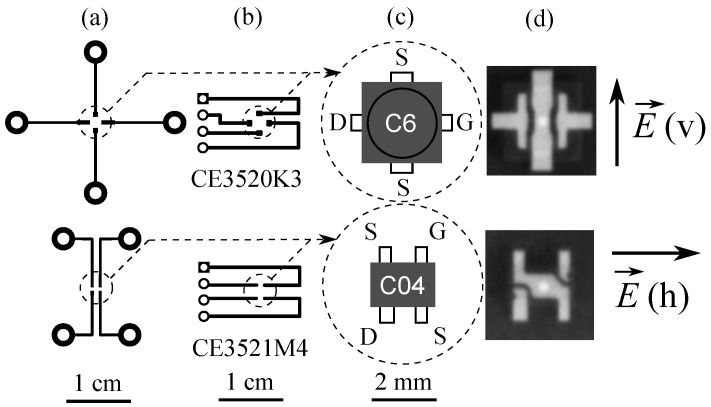
Layouts of printed circuit boards (**a**,**b**), sketches (**c**), and X-ray images (**d**) of the investigated HEMTs where brighter dots indicate the HEMTs’ location areas. Right panel: Vertical (v) and horizontal (h) polarizations of the electric field of an incident THz wave with respect to the relevant PCB configurations.

The layouts of the printed circuit boards (PCBs) and transistor packages are presented in Figure 2. In the first group of PCB designs (Figure 2a), external metal contacts simply serve as extensions of existing contacts in the packages (Figure 2c) with their maintained directions and symmetries. Contact pads for connectors and wire leads are moved away from the expected focal point by at least several wavelengths in this design to prevent their antenna-like influences from being uncontrollable. The second group of PCBs (Figure 2b) was envisaged as the first step in creating one-dimensional relatively dense detector arrays. Therefore, breadboard-compatible 2.54 mm spacing and single-sided connections were implemented. X-ray images of the devices obtained using Acteon X Mind Unity equipment are presented in Figure 2d. The metal contacts inside the package are well visible, and the position of the pHEMT chip is also revealed as a brighter dot in the center. The drain, gate contacts, and chip are placed on the same line in the Micro-X package of CE3520K3, but a more complex arrangement is seen in the case of the 4-pin dual-mold CE3521M4. Therefore, rotated by 90° the drain and gate metal contacts were selected in PCB configuration (Figure 2b) to explore in more detail the possible influence of such a more complex circuitry arrangement.

The presence of epoxy molding compounds (EMCs) in the package generally might affect the performance of encapsulated detectors, and this influence needs to be evaluated. Multiple studies of various EMCs in the THz range [29,30,31] indicated their very similar refractive index values of n=1.975, *n* = 1.897–1.940, and n≈2, respectively. The obtained small variations of the refractive index were attributed to differences in the silica filler content [30]. Such refractive index values can possibly lead to front surface reflectance of the order of 11%, but can also contribute to a more gradual refractive index transition between the free space and the HEMT substrate. The presence of silica in the EMCs might present substantial challenges due to the absorption of THz radiation; however, this absorption is less important at the red end of the THz range. For example, recent measurements of 0.5 mm-thick silica substrates revealed less than 10% absorbance up to 0.7 THz [32]. These reasons allowed us to use the selected HEMTs with factory-provided encapsulation.

The PCBs with soldered-on transistors were mounted on a three-dimensional translational stage system, allowing one to adjust the positions of the detectors. The Spacek GQ-440KS InP Gunn oscillator (Spacek Labs, Inc., Santa Barbara, CA, USA) was used as the source of the 94 GHz radiation, delivering a 42 mW optical power. The THz source was modulated at a 1 kHz frequency using the Agilent 33500B arbitrary waveform generator (Agilent Technologies, Inc., Santa Clara, CA, USA). The gate-to-source DC voltage was adjusted using a Keithley 2400 source meter (Keithley Instruments, Inc., Cleveland, OH, USA), and the detected drain-to-source voltage was recorded by a Signal Recovery 7265 lock-in amplifier (AMETEK, Inc., Berwyn, PA, USA). The step size of the motors was 0.3 mm both in the *x* and *y* axes; the measurement time per pixel was 10 ms and dependent on the time constant of the lock-in amplifier; the measurement time of the whole image with a physical size of 70 × 100 mm (or 250 × 345 px) was in the range of 670 s.

## 3. Results and Discussion

**Figure 3:**
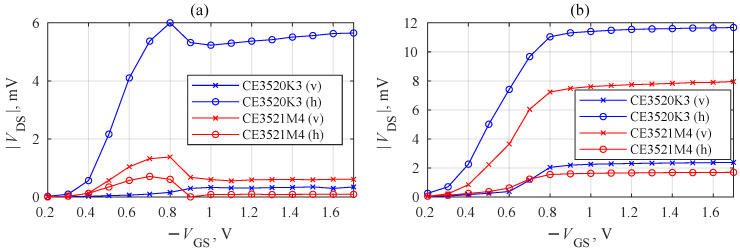
Dependence of detected drain–source voltage $V_{\mathrm{DS}}$ on the gate–source voltage $V_{\mathrm{GS}}$ under the 0.094 THz focused beam. The subplot labels (**a**,**b**) also correspond to the PCB configurations in Figure 2.

The measurement results obtained at 94 GHz are presented in Figure 3a,b. Initially, for each measurement, the three-dimensional translation stages were adjusted to maximize the detected signal from the transistors at a $V_{\mathrm{GS}}=-0.80$ V gate-to-source voltage. Afterwards, the dependence of the detected rectified $V_{\mathrm{GS}}$ drain-to-source voltage was measured as a function of $V_{\mathrm{GS}}$. Two polarizations of the incident THz wave were tested. The letters “v” and “h” indicate vertical and horizontal polarizations of the electric field with respect to the images of the packages given in Figure 2c. The subplot labels in Figure 3a,b correspond to the respective PCB configurations presented in Figure 2.

As seen, independently, the on-packageor PCB-type response signal maxima were observed between $V_{\mathrm{GS}}=-0.7$ V and $V_{\mathrm{GS}}=-0.9$ V, corresponding to the region of the transistor channel pinch-off voltages. We associated the origin of the detected signal with self-resistive mixing [5] and radiation coupling through the antenna effects of the printed circuit boards. The signal exposed strongly pronounced polarization dependence in all four investigated cases. The largest response difference was obtained for the CE3520K3 transistor with PCB option (Figure 3a). This effect can be attributed to an inefficient coupling of THz radiation once the electric field is polarized along the single straight, symmetric, and continuous source-to-source metal contact composed of external PCB tracks (Figure 2a) and the single internal metal contact (Figure 2d). The relatively large dispersion of the maximum $V_{\mathrm{GS}}$ values can potentially be caused by the interplay of the PCB layout, the inner structure properties, and the technological peculiarities of the individual HEMTs.

**Figure 4:**
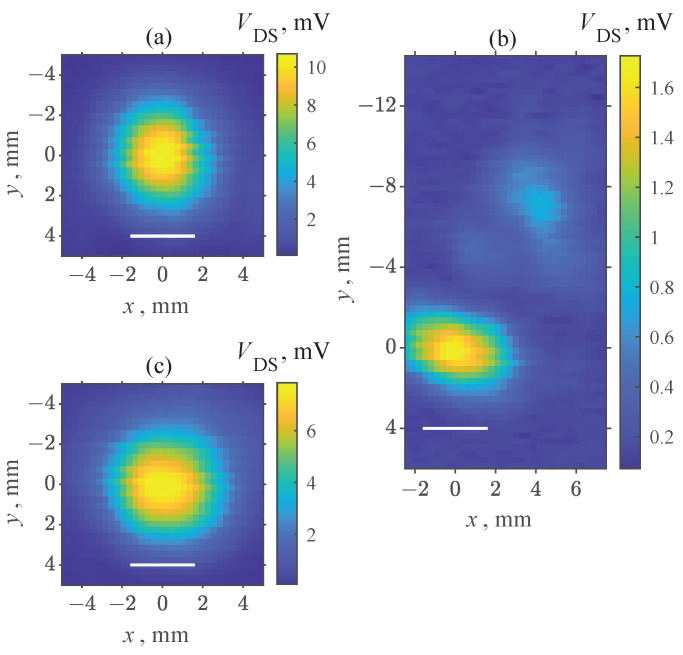
Beam profile images obtained at 94 GHz using: CE3520K3 transistor in PCB configuration “(b)” for horizontal (**a**) and vertical (**b**) electric field polarization; CE3521M4 transistor in the “(b)” PCB configuration for vertical electric field polarization (**c**). One-wavelength-long white lines are shown for an easier comparison.

THz images of the focused THz beam profiles were carried out at the maximal detection voltages for all tested devices. First, the image of the focused beam profile (Figure 4a) was recorded using the CE3520K3 transistor soldered to the PCB of the “(b)” configuration. The electric field was polarized in the drain-to-source direction during this measurement. A single peak was observed with a full-width at half-maximum (FWHM) comparable to the wavelength of the incident THz wave. This result demonstrates that only the HEMT and the closest areas of the PCB mostly contributed to the signal rectification in the case of this most favorable polarization. The influences of beam imperfection and other parts of the PCB were revealed once less favorable source-to-source electric field polarization was selected, and the maximum signal from the detector was reduced by nearly an order of magnitude (Figure 4b). A slightly wider maximum was obtained in Figure 4c when compared to Figure 4a. This suggests that a less symmetric M4 package layout can already play an observable role at these relatively low frequencies. The cross-sectional responsivity of the used HEMTs was found to be around 27 V/W, and the NEP amounted to 510 pW/$\sqrt{\mathrm{Hz}}$ at 94 GHz. The obtained values were in the same range as other commercially available HEMTs [28].

The origin of the detected signal was attributed to the self-resistive mixing [5] and coupling via the antenna effects of the printed circuit boards. THz imaging experiments with this device were repeated under 0.307 THz irradiation to verify this assumption.

**Figure 5:**
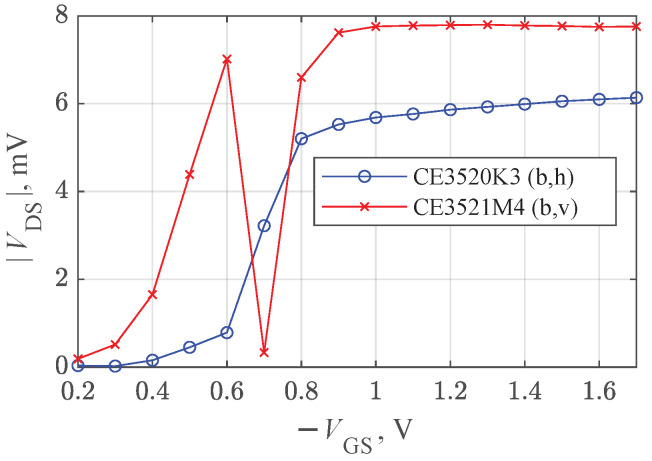
Dependence of the detected drain–source voltage $V_{\mathrm{DS}}$ on the gate–source voltage $V_{\mathrm{GS}}$ under a 0.307 THz focused beam.

For this series of experiments, the Spacek GQ-440KS source was replaced with the Virginia Diodes AMC346 amplifier multiplier chain (AMC), delivering a power of 15.5 mW. The devices were re-positioned to obtain the maximum signal at the focal point at $V_{\mathrm{GS}}=-0.8$ V, and the measurements were repeated. The results are presented in Figure 5. Similar to the previously observed signal (Figure 3b), and increasing pattern can be seen for the CE3520K3 transistor at approximately twice the lower values. However, a substantial reduction in amplitude was observed for the CE3521M4 transistor at 0.7 V. In addition, the signal phase detected by the lock-in amplifier gradually changed from $-32^{\circ }$ at 0.6 V to $147^{\circ }$ at 1 V, indicating the influence of the radiation-coupling-related effects. Such signal changes support the idea that the interaction of the two different detected signals—self-resistive mixing and coupling via the antenna effects of the printed circuit boards—might be involved in the effect.

**Figure 6:**
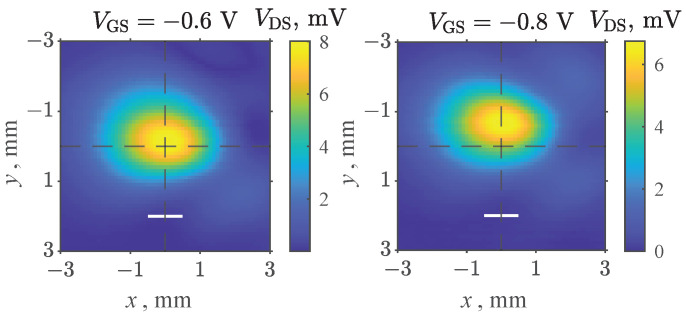
Focused 0.307 THz beam profile images obtained at $V_{\mathrm{GS}}=0.6$ V and $V_{\mathrm{GS}}=0.8$ V voltages applied to the CE3521M4 transistor. One-wavelength-long white lines are shown for easier comparison.

The recorded images of the focused beam at 0.307 THz presented in Figure 6 revealed single response maxima similar to Figure 4. As the obtained spot size was very close to the dimensions of the package, this suggests that the THz radiation was efficiently coupled only when the beam overlapped with the transistor package. At the same time, shorter focusing in comparison with previous measurements’ wavelength, and therefore, tighter, revealed even finer details about the device’s operation. Only the voltage $V_{\mathrm{GS}}$ was adjusted between these two measurements; therefore, a visible vertical shift of approximately 0.4 mm indicated that different parts of the detector dominated at different voltages in forming the signal. Such a large spatial shift is consistent with more effective radiation coupling via different bonding wires or PCB contacts at different voltages. The THz optical system remained stationary during this experiment, and only the position of the HEMT was adjusted by the motorized stages. Therefore, the more sensitive part of the device shifted in the direction from the gate contact side to the drain contact side once the voltage increased.

Since the detected signal values were within the range of mV, therefore, it can successfully be applied for THz imaging.

**Figure 7:**
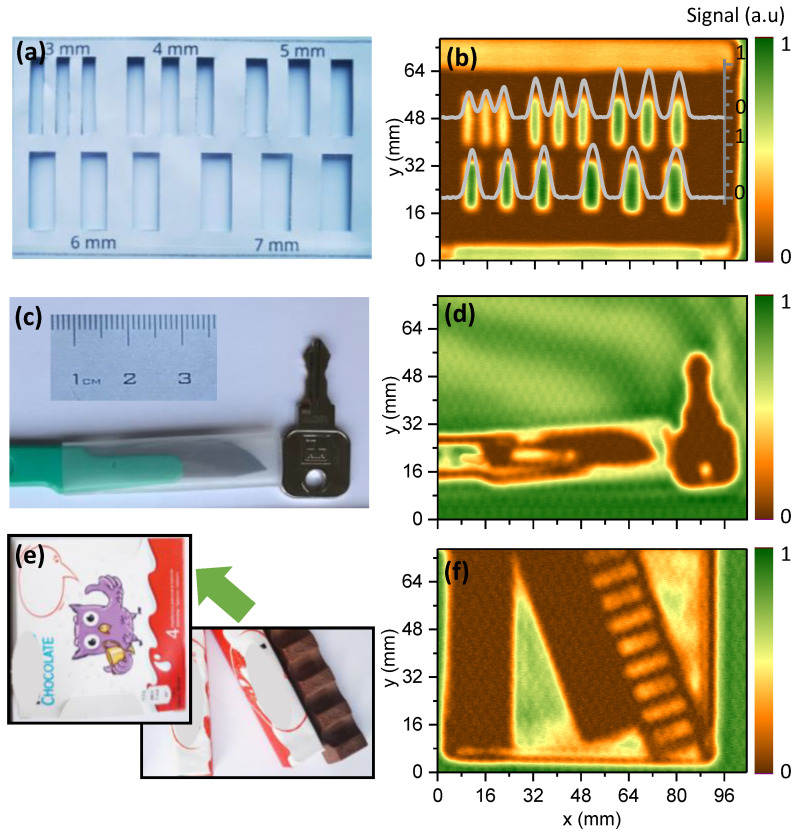
Photos (**a**,**c**,**e**) of several objects and their 94 GHz images (**b**,**d**,**f**), obtained using the prototype low-cost imaging system in the transmission mode of operation. The chocolate image exhibits a contrast of about 30; the knife image contrast reaches about 50, while that of the target amounts to 80; all the values were averaged from at least 1500 pixels.

A low-cost prototype imaging system containing a InP Gunn diode, a paraffin lens, and a CE3521M4 HEMT detector was employed in several THz imaging experiments (Figure 7). The first experiment was related to the common airport or postal security applications—a search for metallic objects. A key and a knife (Figure 7a) were placed in an opaque paper envelope, which was imaged to clearly reveal the shapes of both metallic objects, as is visible in Figure 7b. Even the hole of the key can be distinguished despite being of comparable dimensions to the wavelength of the used THz radiation.

The second experiment was dedicated to illustrating the possibility of imaging system usage in food-industry-related applications. It can be seen in Figure 7c–f. The THz image (Figure 7d) of a partially wrapped (Figure 7c) set of chocolate bars revealed a higher THz transparency in areas of the bars with filling, while the chocolate-only sidewalls remained nearly opaque together with a foil wrapping.

The next experiment was carried out with chocolate bars packed in paper packaging, which is opaque in the visible range (Figure 7e). The positions of the bars and a clear difference between those wrapped in foil and the “bare” bars are visible in the THz image (Figure 7f). Higher-transparency regions with a filling of thinner chocolate can clearly be distinguished without the obstruction of the paper packaging. It is worth noting that, in the imaging setup, we reached a contrast value up to 19 dB, which is sufficient for object recognition in captured images. This is comparable to the THz imaging system presented in [33], where the system signal-to-noise ratio was 20 dB in the transmission geometry at a 213 GHz frequency. For comparison, THz imaging systems equipped with the state-of-the-art CMOS detector can provide an SNR up to 50 dB at a 200 GHz frequency [22]; however, there are few applications that require such a huge system SNR, but simultaneously, this significantly increases the total cost of THz imaging system. It is worth noting that additional contrast enhancement can be reached by increasing the number of scanned images and applying averaging or a mathematical deconvolution technique [34] without an increase in the price of the THz imaging system. Regarding the spatial resolution, it suffered no changes and remained in the range of the wavelength.

Despite the fact that the aforesaid values were not high, it is worth noting that the market price of the used transistor is below EUR 2 for a single unit and even below EUR 1 for larger batches at the time of the manuscript writing. At the same time, specialized commercially available microbolometric THz detectors similar to the ones presented in [13,35] are usually offered at prices of the order of thousands of EUR. Multiple highly sensitive TeraFET detectors can be obtained in a single foundry run [23], but typical costs for such a production batch, as a rule, are much higher than the off-the-shelf devices considered here.

Taking into account the low cost of the paraffin for lens fabrication, the costs of the radiation sources are expected to become a major part of the total system costs. While research-grade 0.1 THz sources might have a cost of the order of thousands of EUR, it can be observed that complete systems on a chip (SoCs) for 77 GHz to 79 GHz automotive radars can cost below EUR 40. These frequencies are only approximately 15% lower than the 94 GHz used in our lower-frequency THz imaging experiments. Therefore, only a small loss of resolution could be expected if this commercially available technology would be reused. Furthermore, research and development work are also being carried out to employ the 122.5 GHz industrial, scientific, and medical (ISM) band for automotive radar applications [36]. Hence, one can infer that even extremely inexpensive detectors and low-cost optics can provide THz images of rational quality and can successfully be implemented in various nondestructive inspection systems. Typical off-axis gold-coated parabolic mirrors of 2 inches in diameter used for the highest optical efficiencies of THz optical systems can cost several hundreds of EUR per piece. Polytetrafluoroethylene (PTFE) lenses of a similar diameter are usually used when higher losses are acceptable and cost in the range of tens of EUR per piece. The application of paraffin lenses requires no expensive materials or machining and display the potential to replace these costs with the nearly negligible cost of paraffin.

Pathways to lower costs can also be envisaged in cases of lock-in amplifiers and x−y positioning systems. For example, it was demonstrated that a custom lock-in amplifier could be built for less than EUR 100 [37], with integrated circuit parts totaling less than EUR 30. The wavelengths of THz radiation define the fundamental limits of the minimum spot size in the mm range achievable at the red end of the THz spectrum. Therefore, a positioning accuracy of 0.1 mm can be found as sufficient and achievable using commercially available off-the-shelf stages for computer numerical control (CNC) applications, such as 3D printing or milling. Such stages are generally available at a single unit cost starting from approximately EUR 125 per axis, including stepper motors. Complete 3D printer systems can be found on the market at a cost below EUR 200 and, with open-source firmware, can serve as another source for low-cost positioning systems.

## 4. Conclusions

The design and performance of an extraordinary low-cost compact terahertz imaging system were demonstrated. Relying on a InP Gunn diode emitter, paraffin wax optics, and the sensing abilities of commercially available GaAs high-electron-mobility transistors with a gate length of 200 nm, THz images of reasonable quality at 94 GHz and 0.307 THz were recorded at room temperature. The detected signal induced by self-resistive mixing and coupling via the antenna effects of the printed circuit boards allowed reaching a system cross-sectional responsivity of 27 V/W and a noise-equivalent power of 510 pW/$\sqrt{\mathrm{Hz}}$ at 94 GHz, thus enabling a spatial resolution in the range of the radiation wavelength (∼3 mm) and an image contrast of nearly two orders of magnitude. Although the emitter accounted for nearly EUR 3000, in contrast, the paraffin wax lens expenses did not exceed EUR 1, and the price of the 20 GHz high-electron-mobility transistors serving as the sensors was in the order of EUR 1/unit. The proposed design opens a promising avenue for an essential reduction in the price of THz imaging systems while still keeping reasonable quality in THz image recording.

## Data Availability

The data used for this study are available from the authors upon reasonable request.

## Abbreviations

The following abbreviations are used in this manuscript:

AMC	Amplifier multiplier chain
DC	Direct current
EMC	Epoxy molding compound
FET	Field effect transistor
FWHM	Full-width at half-maximum
HEMT	High-electron-mobility transistor
NEP	Noise-equivalent power
PCB	Printed circuit board
pHEMT	Pseudomorphic high-electron-mobility transistor
THz	Terahertz
